# Long noncoding RNAs: new players regulating vascular calcification? **Re: Bao S, et al. Genome-wide identification of lncRNAs and mRNAs differentially expressed in human vascular smooth muscle cells stimulated by high phosphorus. Ren Fail. 2020;42: 437–446**

**DOI:** 10.1080/0886022X.2020.1776734

**Published:** 2020-06-18

**Authors:** Tadashi Yoshida

**Affiliations:** Apheresis and Dialysis Center, Keio University School of Medicine, Tokyo, Japan

Dear Editor,

I read with great interest the article entitled ‘Genome-wide identification of lncRNAs and mRNAs differentially expressed in human vascular smooth muscle cells stimulated by high phosphorus’ by Bao et al. [1] The authors conducted microarray analyses and showed that 379 mRNAs and 728 long noncoding RNAs (lncRNAs) were differently expressed in high phosphorus-induced calcified smooth muscle cells (SMCs), as compared to control SMCs *in vitro*. The study has potential implications for the understanding of the molecular mechanisms of vascular calcification in patients with chronic kidney disease (CKD), but I have some concerns on this study.

First, in the high phosphorus-stimulated group, RNAs were extracted from cultured human SMCs treated with high phosphorus conditions for 7 days. This suggests that most of mRNAs and lncRNAs identified in the study are unlikely to play a role in the phenotypic switching of vascular SMCs into osteogenic cells, because cells have already finished the process of trans-differentiation by the time of the harvest of RNAs [2,3]. Rather, they are just a list of genes differently expressed between SMCs versus osteoblast-like cells. To provide a novel insight into the mechanisms of high phosphorus-induced vascular calcification in CKD, a list of mRNAs and lncRNAs induced or suppressed by high phosphorus at earlier time points is required. I believe that findings of the study would be more informative, if temporal changes of mRNAs and lncRNAs by high phosphorus stimulation had been clarified.

Second, although a huge number of mRNAs and lncRNAs were identified in the study, their roles remain undetermined. The authors did not perform any gain-of-function or loss-of-function experiments at all. These experiments should be executed in future. Nevertheless, it should be noted that the NF-κB pathway was identified to be associated with some lncRNAs induced by high phosphorus conditions, as determined by the lncRNA transcription factor mRNA network analysis. Consistent with this, results of previous studies showed that SMC-selective inhibition of NF-κB reduced high phosphate-induced vascular calcification in CKD mice [4]. Clarification of the precise mechanisms whereby lncRNAs interact with NF-κB to promote vascular calcification in CKD is needed.

Finally, the authors only used a single human cultured SMC line for the study. However, in the field of SMC biology, it is sometimes true that the findings obtained in cultured cell systems do not recapitulate what happens *in vivo* [5,6]. It is of critical importance to determine whether the mRNAs and lncRNAs identified in the study are also regulated in animal models of vascular calcification *in vivo* and/or in human CKD patients. In addition, there is also clear evidence that vascular SMCs have diverse embryological origins [5,6]. Most vascular SMCs are derived from local mesoderm populations. However, the major arteries in the head and neck region contain a significant fraction of cells derived from the neural crest that plays a key instructive role in the complex morphogenesis of brachial arch-derived vessels. Moreover, SMCs in the coronary arteries are derived from the proepicardial origin. It is interesting to determine whether any SMCs derived from multiple embryological origins utilize common molecular mechanisms for vascular calcification in future.
